# The Pharmacy Game-GIMMICS^®^ a Simulation Game for Competency-Based Education

**DOI:** 10.3390/pharmacy8040198

**Published:** 2020-10-24

**Authors:** Tanja Fens, Claudia M. Dantuma-Wering, Katja Taxis

**Affiliations:** 1 Department of PharmacoTherapy, -Epidemiology and -Economics, Groningen Institute of Pharmacy, School of Science and Engineering, University of Groningen, Antonius Deusinglaan 1, 9713 AV Groningen, The Netherlands; c.m.dantuma-wering@rug.nl (C.M.D.-W.); k.taxis@rug.nl (K.T.); 2 Department of Health Sciences, University of Groningen, University Medical Center Groningen, Hanzeplein 1, 9713 GZ Groningen, The Netherlands

**Keywords:** pharmacy, education, serious-game, simulation, the pharmacy game, GIMMICS

## Abstract

The profile of the profession of pharmacists has profoundly changed over the last decades. Pharmacy education has moved towards competency-based education. The pharmacy game, called GIMMICS^®^, developed at the University of Groningen, is unique in combining simulation with serious gaming to teach a wide range of competencies. In this article, we describe the learning goals, the assessment methods, the teaching tools, and the students’ view of the pharmacy game. The learning goals are to train the competencies of collaboration, leadership, communication, and pharmaceutical expertise. The core of the game is the simulation of community pharmacy practice activities, such as patient counseling, processing of prescriptions, and collaboration with other health professionals. Students are assessed individually and as a pharmacy team. The pharmacy team, with the largest number of patients wins the game. Student evaluations show that they value the course. Currently, seven universities from around the globe have adopted the pharmacy game in their curriculum, adjusting the course to their country’s pharmacy practice and educational system.

## 1. Introduction

The profession of pharmacy has profoundly changed over the last decades. The role of pharmacists in community and hospital practice has expanded from product dispensing to patient care and medicine management [1]. Those developments have led to changes in the pharmacy curricula, learning goals, and teaching methods in many universities around the world [2,3,4]. Pharmacy education has moved towards competency-based education [5,6,7,8], and several different frameworks have been developed to describe the competencies of pharmacists [7,9]. Since 2006, the Canadian Medical Education Directives for Specialists (CanMEDs) model is used in the Netherlands to describe the seven competencies of the Dutch pharmacists [10], and this model has been adopted in all Dutch schools of pharmacy [11].

Many teaching methods are used in pharmacy education, but simulation-based training and the use of a serious game have gained particular attention [12,13,14,15,16,17,18,19,20]. Simulation is understood as an artificial representation of a real-world process to achieve educational goals through experiential learning [21,22]. It has been shown to be an effective teaching method, compared to traditional methods used in pharmacy [12]. In particular, studies showed that students gain self-confidence [23,24], are motivated to learn [25], and improve their professional skills [24] when simulation methods are used. Students liked the method and were satisfied with the teaching [25]. Simulation methods can be combined with a serious game [26]. Serious game in education means to specify a game set-up, game rules, and activities where students compete with each other [27,28]. Combining simulation with a serious game allows students to be active participants in the teaching, apply their knowledge, gain knowledge and practice various skills by analyzing situations, interacting with consumers, patients, health professionals, and making decisions in a safe environment [13,29,30]. The active participation generates a memorable experience which serves to recall easier the learned material [31]. At the University of Groningen, we developed the pharmacy game GIMMICS^®^ [32] about twenty years ago. It combines simulation with a serious game. The aim of this article is to describe the learning goals, the assessment methods, the teaching tools, and the students’ views of the pharmacy game, GIMMICS^®^.

## 2. The Pharmacy Game, GIMMICS^®^

The pharmacy game, GIMMICS^®^ teaches pharmacy students by simulation of a health care environment using a serious game [32]. This game has evolved over the last twenty years at the University of Groningen. Moreover, its concept is currently adopted in six other universities, University of Utrecht, the Netherlands, Free University of Brussels, Belgium, University of Nottingham, Great Britain, Griffith University, Australia, University of Bath, Great Britain, and the University of Vilnius, Lithuania.

In the Netherlands, the study of pharmacy consists of a 3-year Bachelor of Science in Pharmacy, followed by a 3-year Master of Science in Pharmacy. The pharmacy game, GIMMICS^®^ is a compulsory and standalone course in the last year of the master [11]. It accounts for eight European Credit Transfer and Accumulation System (ECTS) points. The course is usually organized three times-a-year. Each time, between twenty and thirty-six students participate. Its duration is five weeks. The course is organized in fourteen “study days” comprising of lectures, self-study, and group work, and eleven “practical days” when students work in their own pharmacies as pharmacists or pharmacy staff [32]. This approach combines theoretical with practical experience to enhance learning [33,34]. Appendix A gives an overview of the course structure and daily activities. 

At the start of the course, students are divided into five to six teams responsible for setting up and running their own simulated community pharmacy. They develop a business plan, including mission and vision for their pharmacy and establish a quality management system. They can also choose their own area to specialize, e.g., in polypharmacy or diabetes. Students have to reflect on their own competencies in a written self-reflection essay, which is an individual assignment. Pharmacy teams also have to perform different types of professional activities, as outlined below. All teaching activities are facilitated through a dedicated software solution, which is available online. This software is used to generate and manage the activities for the students and display their individual and group achievements and scores [32,35].

### Game and Simulation Elements in the Pharmacy Game

The game is set up in the virtual village Pildorp with 40,000 inhabitants, where each pharmacy is located in a specific neighborhood. At the start of the game, each pharmacy is assigned 8000 patients. Depending on the performance of the pharmacy team, they gain or lose patients throughout the game. The team that ends the game with most patients is the winner of the game [32]. 

Pildorp has a mayor who opens the pharmacies at the beginning of the game. The virtual village also has a web-based newspaper where students and staff may publish announcements and activities, such as a thematic evening for medicine use during pregnancy, advice for medicine intake during religious holidays, and fasting, or advice about the pharmacogenetic profile of a patient and the use of medication. The simulated pharmacies are set up in usual classrooms equipped with computers that run one of the Dutch community pharmacy information systems to register patients, their medication history, and perform medication monitoring, such as medication-medication interaction checks, print medication labels, etc. The computers have internet access to run the game software and other programs for emailing or literature searches. Rooms have a telephone, printers, some office furniture, such as tables, chairs, and shelves. Furthermore, students have a large supply of empty boxes of medicines, medical devices, and other materials to equip their pharmacies. 

During the course, pharmacy teams encounter many simulated situations. This includes interactions with patients, played by actors (university staff, former pharmacy students, trained volunteers, and professional actors). Each game actor gets assigned one case, and they play this case in each pharmacy, which takes between two and three hours. About thirty-three actors participate in one game, on average, three actors per day. Actors (outside university staff) receive a small fee for their service, as well as reimbursement of their travel costs. Other simulated situations include communications with general practitioners (GPs) played by real GPs or GPs in training. During the game, on the so-called eleven practical days, two GPs are communicating with the students, every two hours per day. Furthermore, situations, such as the visit of inspectors or medical representatives, played by university staff or real-life representatives from different organizations, are also simulated during the game.

## 3. Learning Goals and Activities

### 3.1. Learning Goals

The learning goals of the pharmacy game are in line with the roles and competencies of pharmacists described in the CanMEDs model [8], particularly focusing on collaboration, leadership, communication, and pharmaceutical expertise. Some of the activities around these roles are illustrated in Table 1.

#### 3.1.1. Collaboration

Setting up the teams of students to run one pharmacy is an important part of learning to collaborate. Students have to decide who is the manager, and divide the roles and tasks of the pharmacy staff amongst themselves, i.e., who-does-what and when [36]. Furthermore, they have a teambuilding day to strengthen the team spirit and establish better relationships. During this activity students’ personality is being examined using the DiSC test [37]. Each pharmacy team has a coach (a member of staff) to help them understand the underlying problems within the team, but also to point out how to optimize the collaboration. Furthermore, students have to collaborate with other health care professionals, such as GPs and doctor’s assistants, consulting them about prescriptions, problems of patients, and some more complex cases (e.g., the supply of medicines for a patient requesting euthanasia). Students also have to interact with staff of other institutions or companies, such as hospitals, franchise companies, pharmaceutical compounding companies, or health-insurance companies. Hospital staff are mostly contacted during working hours to enquire about prescriptions of patients discharged from hospital. Students also arrange the supply of pharmacy-based preparations (magistral preparations), as they are not equipped to prepare medication in the simulated pharmacies. Also, each pharmacy team has to set up contracts with one or more health-insurances of their choice.

#### 3.1.2. Leadership

Working in teams gives a perfect environment to practice leadership [38], for example, as the pharmacy manager. Students may take turns being the pharmacy manager. The focus is on training the following skills:

- Leading from a vision;
- Coaching and managing individuals;
- Taking responsibility;
- Entrepreneurship and innovation.

Students get several lectures on personal leadership. They have to reflect on their leadership skills in their self-reflection assignment, as well as to give each other feedback on the performance within their team. The basics of these skills can be trained in several situations. For example, deciding on a vision in the business plan, processing of the prescriptions on time, or organizing the communication with external parties. Once or twice in the game, emergency situations, such as flooding of the pharmacy building or a pandemic, may arise. Leadership skills are needed to handle such situations. In routine situations, leadership skills are trained by initiating, executing, and supervising projects. Moreover, showing confidence while counseling patients is also a way of taking the leading role. Another activity to train leadership is interviewing and hiring pharmaceutical assistants/technicians, played by pharmacy assistants/technicians in training. This activity is facilitated in collaboration with a school of pharmaceutical technicians. 

#### 3.1.3. Communication

This section is closely related to collaboration. We have already pointed out with whom students communicate to establish collaboration (health professionals and other professionals) in many different situations. Face-to-face, telephone, and email communications are trained, as well as group situations, such as the presentation for a group of GPs during a pharmacotherapeutic consultation meeting or other pharmacists. Furthermore, students get to interact with several patients per day. The patients are not always straightforward requesting a medicine or their prescription, but also asking for advice, a second opinion, do not speak the local language [39], are unsatisfied, angry, or happy. Also, a “patient” can be a mystery shopper, such as a quality inspector, who attempts to get medicines without a prescription. All of the situations are provoking proactive thinking and doing, but also enhance the ability to handle diverse situations [24].

#### 3.1.4. Pharmaceutical Expertise

Being involved in multiple activities, students face the real challenges of pharmaceutical professionals. During the course, students also have “study days” covering the core areas in pharmacotherapy. During the practical days in the simulated pharmacies, the focus is on pharmaceutical care. Practicing pharmaceutical expertise is mostly achieved by assessing prescriptions, detecting medication errors, and discussing the appropriateness of prescribing with GPs or other health professionals and patients. In patient encounters, students have to ask the right questions to identify medication-related problems and give adequate advice to solve problems.

### 3.2. Organizing the Practical Teaching

**Figure 1:**
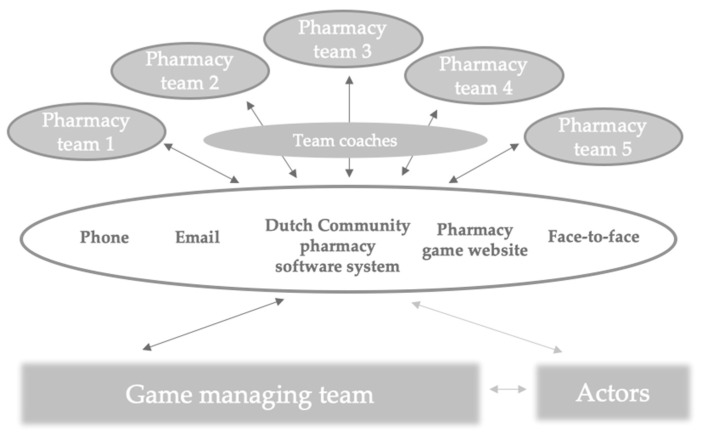
Structure of the pharmacy game GIMMICS^®^. Students are situated in the pharmacies, representing a pharmacy team. Each pharmacy team has a team coach assigned to them. The interactions between pharmacy teams and the game managing team are made through the facilitating tools and media presented in the middle of the figure. Actors play patients visiting the pharmacies and assess their pharmacy visit to the game managing team.

A game management team comprising of one lecturer, one assistant, and a student assistant is leading the teaching and coordinating the processes in the “practical days”. In the “study days”, various lecturers are covering different topics (described in Appendix A). Also, each pharmacy team is assigned with an independent team coach supporting collaboration and reflective activities. The communication between students and the game management team is facilitated with the phone, email, national community pharmacy software system, the pharmacy game-website, as well as face-to-face communication (Figure 1). 

## 4. Assessments

**Figure 2:**
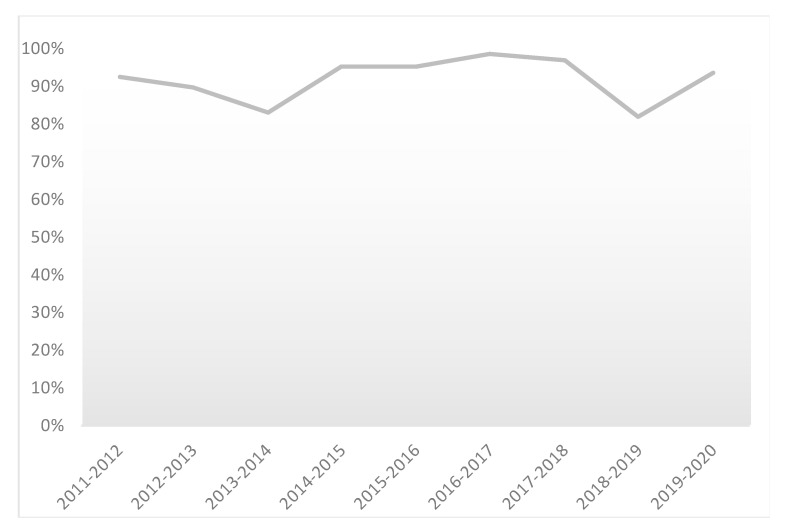
Percentage of students successfully accomplishing the course of the pharmacy game, GIMMICS^®^ presented from the academic year 2011–2012 up to 2019–2020.

Over the last nine academic years, on average, 92% of the students passed the course, ranging between 99% and 81%. These results are displayed in Figure 2. If students fail the course, they rarely have to play the whole game again, but they may get individual assignments for areas with low performance. 

All the different activities that the pharmacy teams have to perform are assessed using assessment sheets with pre-defined criteria. Based on the assessments, pharmacy teams lose or gain points/patients each day of the game. The minimal score to pass as a team is to have nine thousand patients at the end of the game (while each team enters the game with eight thousand patients). As highlighted above, the winner is the team with the highest number of patients. The best performing pharmacies achieve about ten thousand patients. However, this varies per game, as each game is unique in the mix of cases played, and the pharmacy teams having different ideas of running a pharmacy. Students often receive relatively low scores for patient cases involving an angry patient, and receive relatively high scores for a well-developed business plan. 

Each student is also assessed individually. The individual scoring is based on assessments of the communication skills, peer assessments for leadership, and collaboration, as well as their assignment of self-reflection. The minimum score for a student to pass is achieving 55% for each competence, as described in the *learning goals* section. Students get a pass or fail for the course. A detailed description of each individual and group assessment activity, points/patients gained or lost, an association to the learning goal, as well as frequency/quantity per activity is given in Table 2. 

### 4.1. Example of a Patient Case

To illustrate more details about playing the game and the assessment procedure, we selected a typical patient case. One actor plays this case in all the pharmacies, and after visiting each pharmacy, completes two assessment forms, one for pharmaceutical expertise and one for communication. Students gain points/patients individually for both competencies. The group-score is the sum of those scores.

#### 4.1.1. The Case

The actor visits the community pharmacy and asks if they can do something about the menstrual complaints of her 16-year-old daughter. She suffers from pain and cramps in her lower abdomen and has a headache. She has had this since the start of her menstruation. The complaints are not so severe that she has to report sick from school, but she prefers not to do sports or meet friends because of the complaints. Menarche (1st menstrual period) was at the age of 13. Since then, the pain has been the same in every menstrual period. It starts just before menstruation and lasts for 2–3 days. Further, the cycle is regular and lasts 28 days. She sometimes takes paracetamol, but that hardly helps. No further medication. She now wants better pain relief to get through her periods better.

This case refers to the clinical problem of primary dysmenorrhea in women under 25 years. The pharmacist is supposed to ask questions about the girl’s symptoms, in particular, the duration and intensity of the pain and the timing of the pain in relation to the menstruation; the duration and intensity of the bleeding, and the presence/absence of other symptoms, such as fever. This is to distinguish between primary dysmenorrhea (usually in a woman under 25 years of age) and secondary dysmenorrhea (inflammatory disease, sexually transmitted diseases, endometriosis, or myoma; usually in women >25 years) or other causes, such as an ectopic pregnancy [40]. 

This case is estimated to take about ten minutes per pharmacy. 

#### 4.1.2. The Assessment

The actor is asked to complete two scoring forms. One form is to assess pharmaceutical expertise. In this case, it is first assessed whether students are able to identify the clinical problem by using the WWHAM model (who, what, how, and medications) and ALECOBO model (nature of complaints, localization, seriousness, chronology, originate, influencing, opinion). Secondly, it is assessed whether the correct advice was given. In this case, whether the pharmacist explained, for example, that pain during menstruation is common as about 60 to 80% of young women between the ages of 12 and 24 suffer from it. It is important to assess whether the pharmacist was able to distinguish between primary (<25 years) or essential dysmenorrhea (without demonstrable underlying pathology) and secondary dysmenorrhea (with the underlying disease). Good non-pharmacotherapeutic advice is to apply heat locally, like a warm water bag or jug, approximately 39 °C. Pharmacotherapeutic advice includes dispensing non-steroidal anti-inflammatory drugs (NSAID), e.g., naproxen or ibuprofen in adequate doses, taken at the start of the symptoms and continued for the expected duration of the symptoms (usually two to three days), mentioning that paracetamol is not the first choice for menstrual pain, but can be added to the NSAID [41]. Furthermore, hormonal contraception may be considered, which requires referral to the GP. 

The actor also completes a second scoring form to assess verbal/non-verbal communication, structure, and duration of the patient encounter. Criteria that are scored include how the student opens the conversation (greeting, introduction, attitude and empathy and focus on patient/client); how the student performs during the middle part of the conversation, i.e., problem identification/counseling (logical flow, active listening, connecting with the patient, paraphrase and/or emotional reflection, providing space to ask questions and tell a story) and finally how the student is able to close the conversation (summary, repeating agreements to ensure the instructions are understood). 

It is important to note that students do not receive those assessment forms (or any of the other scoring forms) until after the whole game is completed. They only receive the overall score per case/assignment (continuously uploaded online on the game-web page), i.e., the number of patients that the pharmacy team gains or loses per case is +/−20 patients fixed score for communication, and as many patients for pharmaceutical advice on the case content. 

## 5. Tools and Functionalities

### 5.1. Online Operational Tool

The main tool for running the game is the webpage of the pharmacy game (pharmacygame.education). The software of this web was created to facilitate the activities during the game played in Groningen, which further was expanded to be used by multiple universities. 

The software is used for general game management activities, such as registering the students per pharmacy team, entering the results of individual and group assessments. General information over game setting, game rules, and game schedules are presented as well. The software also displays the scoring of each pharmacy team over time, so students can compare their performance. Furthermore, the game’s “newspaper” is online, allowing students and staff to publish news and pictures as part of the game.

Essential for the management of the game are the following three programs: 

#### 5.1.1. Case Management System (CAMS)

The Case Management System (CAMS) is used to manage all the patient cases that are possible to play within the game. All information on the case is structured as: Unique code, title, administrative data, such as the author of the case, the theme, the purpose, the learning goal and the time to play the case, information on the patient (demographic data, medical history, and medication history), instructions for actors/patients and in some cases also for students. In addition, information that may be included in the “possibility”, an indication of what is possible or what is expected from one particular case and remarks from the authors/game managing team. The system is set up to easily edit cases, search for cases, or browse the collection of cases. During the game, the CAMS system is used to assign cases to specific actors and generate a description of the case. Such descriptions include the assessment forms for the actor to print out for use or consult online. 

#### 5.1.2. Actor Registration System (ARS)

The Actor Registration System (ARS) is a database of actors with the registration of names and electronic addresses. The system also includes an interactive agenda to set the time and date of the actors to avoid overlapping of visits to the pharmacy. The game management team uses the system to assign cases to the actors, to send invitations and information on the case to be played. The actors can also access the system to see all the bookings or adjust their profiles. 

#### 5.1.3. Prescription Generator

A prescription generator is a tool that is used by the game managing team to generate all the prescriptions which have to be processed by the pharmacy teams. The information included for every prescription is the information on the prescriber, information on the patient, date, and information on the prescribed medication. The game management team generates the prescriptions and sends them to the simulated pharmacies. Important functionality is the possibility to follow the patient over three months (as in the virtual game, one day counts as one week). Moreover, during the game, the prescription generator is used for communication and collaboration with the GPs concerning the prescriptions. 

## 6. Evaluation of the Pharmacy Game, GIMMICS^®^ by Students

The official evaluation reports from the students (from 2011 up to 2020) show that the course is overall valued by students with a grade of 7.8 out of 10. Students report that the course is well placed in the educational program and well organized. Students report that they feel motivated to learn. They appreciate to apply pharmacotherapy, get a better understanding of the processes of a community pharmacy, improve their communication skills with patients and GPs, train collaboration, practice in a team, and be able to translate innovative developments in practice.

An aspect that is repeatedly reported in the evaluation reports is that students feel they lack feedback about their performance during the course. Many students find assignments supervision poor, and the assessment standards unclear. They would like to receive more direct feedback on their individual and their team’s performance. As highlighted above, students only receive the scores on each case/assignment, but no direct information about mistakes, failures, or successes. They also can compare their performance with the performance of other teams.

Within the game environment, students are supposed to individually and as a team reflect on their performance to identify mistakes, weaknesses, and opportunities to improve. During the game, the pharmacy team has the possibility to use a “wild card” and ask the game management team for direct feedback. At the end of the game, all assessments are available for the students for review, but this is hardly used. This aspect of how we provide feedback to the student in the game reflects the reality as close as possible, and the reality is that when working in a pharmacy, you do not always receive direct feedback.

## 7. Discussion

The pharmacy game GIMMICS^®^ facilitates the transition from a pharmacy student to a pharmaceutical professional by teaching a range of competencies essential for practice [42]. Within this game, the students are taught to be leaders, communicators, collaborators, and pharmaceutical experts. Such competencies are challenging to teach and assess using traditional teaching methods like lectures and written exams. Those competencies can be taught and assessed during internships in community or hospital pharmacies. In contrast to the internships in practice, the pharmacy game provides a completely controlled environment where it is safe to make mistakes and learn from them. Moreover, using the methods of the pharmacy game creates an opportunity for all these competencies to be taught together. The competitive nature of the game stimulates motivation to accomplish the activities to win the game. Leadership skills are learned by prioritizing tasks, organizing a team, and taking responsibility. Collaboration and communication with each other (or with other health workers, institutions, and patients) are trained, and pharmaceutical expertise has to be demonstrated. The students at the University of Groningen value this form of education. This is in line with studies showing that serious games in education and training of health professionals bring additional values in the learning processes [13,29,30]. A recent review described several assessment models used in pharmacy education [5]. The pharmacy game includes elements, such as simulated patients encounter (role-play) [43], Objective Structured Clinical Examination (OSCE) [44], Workplace Based Assessments (WPBA) [45,46], portfolio [47], and Entrustable Professional Activities (EPA) [48]. Yet, a unique aspect of the game is the possibility for individual and group assessments. Part of the individual assessments includes a written self-reflection and peer-reflection. These have been recognized to be essential components to stimulate the life-long learning process [49]. Moreover, the pharmacy game also includes the five levels of the Millers Pyramid, knowledge, competence, performance, action, and identity [50]. 

Despite having twenty years of experience with the pharmacy game, several challenges remain. First, we have not performed controlled studies comparing the learning outcomes of the pharmacy game with other teaching methods using validated instruments. Such studies could present measurable data showing the level of competence improvement or competence gain with each teaching method. A first step would be to develop validated instruments to measure competencies [51]. Furthermore, we provided data from student evaluations, which are part of our University’s quality assurance system. The evaluation forms were changed multiple times through the years, and the extracted data reflects this heterogeneity. Therefore, we only descriptively present the sections included in more than one assessment form. This emphasizes the need for standardization of such evaluations ideally structured together with the students. Along with it, we could perform a more in-depth/qualitative analysis of individual comments and suggestions to capture the student voice in a systematic way.

In the future, we would like to investigate more systematically how this educational concept is used in different universities, emphasize the similarities and differences and bring conclusions that will lead to further improvement of this educational tool. Therefore, more methodologically sound research is needed in pharmacy to advance the development of evidence-based simulation games in pharmacy education further.

## 8. Conclusions

The pharmacy game GIMMICS^®^ as part of an undergraduate pharmacy curriculum brings additional value to the educational program to teach and assess a wide range of competencies.

## Figures and Tables

**Table 1:** Learning goals and examples.

Learning Goal	Examples of Activities from the Game
Collaboration	Students agree on their roles within their pharmacy team (pharmacist/pharmacy assistant) to best run their pharmacy. The coaches help them during this process. Teambuilding activities, supported by the DiSC test. Discussing patient’s prescriptions with a GP or Doctor’s Assistant. Negotiating supply or reimbursement of prescriptions with staff from the hospital, pharmaceutical compounding company, or health-insurance.
Leadership	Processing a large number of prescriptions in a short time. Negotiating tasks among their team. Writing a business plan and an annual report for the pharmacy. Choosing a specialization for their pharmacy in line with their mission/vision. Optimize the quality management system.
Communication	Counseling patients on their medicine use in various clinical cases. Leading pharmacotherapy quality circle meetings with general practitioners. Solving dilemmas and discussing situations with colleagues from their own, but also from other pharmacy teams.
Pharmaceutical expertise	Assessing prescriptions. Identifying patient problems and giving the correct advice. Substantive assessment of a case. Medication review/polypharmacy.

DiSC, Dominance, Influence, Steadiness, Conscientiousness; GP, General Practitioner.

**Table 2:** Activities-assessments per learning goal/competence achieved.

Individual Score Activity	Points/Patients	Competence	Frequency/Quantity
**Communication with GP**	+/−20	2	3–4 assignments per student 2 GPs, 2 h/day each (11 days)
**Actor (counter, telephone, email)**	+/−40	1 & 2	5–6 assignments per student 3 actors, 2-3 h case/day (11 days, 33 cases)
**Euthanasia case requested**	+/−20	2	1 student in each pharmacy addresses the GP request for euthanasia
**Euthanasia case proceed**	+/−40	1 & 2	1 student in each pharmacy attends the GP when coming to collect the euthanasia and discuss final advice
**Case “auction”**	+/−40	1 & 2	1 student in each pharmacy order (form a fictional foundation in Pildorp) materials, such as inhalation, wound care, homeopathy, phytotherapy, and other products that are fitting the mission/vision of their pharmacy
**Evening-, night-, and weekend-service case**	+/−40	1 & 2	2 students in each pharmacy and each pharmacy is called twice
**Medication review patient—meeting 1 (patient)**	+/−20	2	1 student in each pharmacy does medication review in communication with a patient
**Medication review patient—meeting 2/3 (GP/patient)**	+/−40	1 & 2	1 student in each pharmacy does medication review in communication with GP, and once more in communication with a patient
**Team score activity**			
**Working on prescriptions**	+/−60	1	30-40 prescriptions per pharmacy/day (11 days)
**Writing business plan**	+/−100	3 & 4	Once
**Writing annual report**	+/−100	3 & 4	Once
**Pharmacy opening**	+/−40	3 & 4	Once
**Innovation project**	+100	3 & 4	The insurance company of the inhabitants of Pildorp (represented by real pharmacist or entrepreneur) can give one pharmacy a prize of maximum +100 points, for best innovative health-project
**Quality; risk-analyses**	+/−40	3 & 4	Once, the students should perform a risk analysis to increase pharmacy quality. This is done using the quality management system
**Quality; intern audit self-care/pharmacy-based preparations**	+/−40	3 & 4	Once, over self-care and once over pharmacy-based preparations. The student assesses the procedure in the pharmacy in these fields using the quality management system and records data over adherence to the requirements
**Quality; use of the quality management system**	+/−1	3 & 4	Once, gaining 1 point if registering a fault, complain, or a compliment in the quality management system
**Quality; complaints and errors analysis**	+/−40	3 & 4	Once, students should analyze the errors/complaints/compliments with an indication for possible policy change and further needed actions in that regard
**Vacancy text**	+/−40	2 & 3	Once, placing a vacancy for a pharmacy assistant/technician
**Job interviews**	+/−60	4	Once, interviewing a new potential pharmacy assistant/technician
**PAE**	+/−40	1	Optional activity, students may choose from various lectures to attend and gain points for “keeping their licenses”
**FTO preparation**	+/−20	3 & 4	Once, students prepare for the pharmacotherapeutic consultation with GPs
**FTO content**	+/−40	1	Once, consultation-content assessed
**FTO presentation/execution**	+/−40	3 & 4	Once, students attending and actively participating the pharmacotherapeutic consultation with the GPs
**FTO minutes**	+/−20	2	Once, students should provide minutes from the meeting
**Speed dates**	+/−40	3 & 4	Once, student’s meeting with pharmacy-chain representatives
**Small project**	+/−40	3 & 4	Individual-pharmacy projects: Ramadan in the pharmacy, an injection day for diabetics, an information evening for pregnant women, etc.
**Big project**	+/−100	3 & 4	Activities requiring project management: cooperation with a nursing home, cooperation with an asylum seekers’ center, etc.

Competencies: 1—Pharmaceutical expertise; 2—Communication; 3—Collaboration; 4—Leadership GP, general practitioner; PAE, post academic education; FTO, Pharmacotherapeutic consultation with general practitioners in the region. The general practitioners in training (medical students) and the students/pharmacists jointly discuss and determine the pharmacotherapy policy and what is prescribed; "Speed dates" are appointments with pharmaceutical-chain representatives to determine whether students/pharmacists from one simulated pharmacy want to join a pharmacy-chain or ramming an independent pharmacy; "Big project" generally spreads across the entire game and involves more people/activities, while a "small project" is intended for within the own pharmacy and patients.

## Appendix A

**Table A1:** Course schedule, activity and content.

Week	Game-Day	Type of Activity	Content
1	1	Study day	Introduction, mission and vision, business plan
	2	Study day	Pharmacy information system
	3	Study day	Health insurance
	4	Study day	Preparations
	5	Study day	DiSC typology
2	6	Practical day	Practicing day with open hour for questions
	7	Study day	Financing, psychiatry and prescriptions
	8	Study day	Quality Management System
	9	Practical day	Official opening of the pharmacies
	10	Practical day	Prescriptions, cases, and quality Quality Management System in practice
3	11	Practical day	Prescriptions and cases
	12	Practical day	Prescriptions and cases
	13	Study day	Employment
	14	Practical day	Polypharmacy consultations, prescriptions, and case descriptions, and cases
	15	Study day	DiSC typology and teambuilding
4	16	Study day	Leadership, euthanasia, medical devices
	17	Practical day	Polypharmacy consultations, prescriptions, and cases
	18	Study day	Clinical pharmacy and mid-evaluation
	19	Practical day	Job interviews and terms of employment, prescriptions, and cases
	20	Practical day	Polypharmacy, prescriptions, and cases
5	21	Practical day	Prescriptions and cases
	22	Pharmacotherapeutic consultation	Euthanasia and privacy
	23	Practical day	Prescriptions and cases
	24	Final interview	Company representatives visit
	25	Self-reflection	Cases overview with the students and delivering the self-reflection

This table is an example of the course during November–December 2020, at the University of Groningen. Each day accounts for one week in the game. It illustrates the main frame and content of this course. The type of activity reflects if the student is involved in a theoretical or practical educational activity, while the content reflects the main topic covered in the activity.

DiSC-Dominance, Influence, Steadiness, Conscientiousness; DiCS typology within this game means assigning colors (red, yellow, green or blue standing for dominance, influence, steadiness, and conscientiousness accordingly) among the students reflecting teamwork and collaboration.

All the theoretical activities are based on post-academic education “PAO-Farmacie” that offers education focused on quality, topicality, and practice.
